# The effects of head movement on dual-axis cervical accelerometry signals

**DOI:** 10.1186/1756-0500-3-269

**Published:** 2010-10-26

**Authors:** Ervin Sejdić, Catriona M Steele, Tom Chau

**Affiliations:** 1 Bloorview Research Institute, Holland Bloorview Kids Rehabilitation Hospital, Toronto, Ontario, Canada; 2 Institute of Biomaterials and Biomedical Engineering, University of Toronto, Toronto, Ontario, Canada; 3 Division of Gerontology, Beth Israel Deaconess Medical Center, Boston, MA, USA; 4 Harvard Medical School, Harvard University, Boston, MA, USA; 5 Toronto Rehabilitation Institute, Toronto, Ontario, Canada; 6 Department of Speech-Language Pathology, University of Toronto, Toronto, Ontario, Canada

## Abstract

**Background:**

Head motions can severely affect dual-axis cervical acceloremetry signals. A complete understanding of the effects of head motion is required before a robust accelerometry-based medical device can be developed. In this paper, we examine the spectral characteristics of dual-axis cervical accelerometry signals in the absence of swallowing but in the presence of head motions.

**Findings:**

Data from 50 healthy adults were collected while participants performed five different head motions. Three different spectral features were extracted from each recording: peak frequency, spectral centroid and bandwidth. Statistical analyses showed that peak frequencies are independent of the type of head motion, participant gender and age. However, spectral centroids are statistically different between the anterior-posterior (A-P) and superior-inferior (S-I) directions and between different motion. Additionally, statistically different bandwidths are observed for head tilts down and back between the A-P and the S-I directions.

**Conclusions:**

These differences indicate that head motions induce additional non-dominant spectral components in dual-axis cervical recordings. The results presented here suggest that head motion ought to be considered in the development of medical devices based on dual-axis cervical accelerometery signals.

## Background

Patients living with the effects of stroke and patients with neurodegenerative illnesses often have swallowing difficulties (dysphagia) [1]. The videofluoroscopic swallowing study (VFSS) is the current gold standard for the detection and management of dysphagia [2]. Nevertheless, excessive exposure to ionizing radiation, long waiting lists for the radiology suite at hospitals and the lack of appropriate equipment at every hospital render VFSS infeasible for ongoing patient monitoring [3,4]. In recent years, a technique involving the attachment of an accelerometer to the patient's neck conveniently termed cervical accelerometry emerged as a supplemental approach for the noninvasive assessment of swallowing disorders [5]. Traditionally, single-axis accelerometers were used [6]. However, recent studies have shown that dual-axis accelerometers yield more information and enhance analysis capabilities [7,8], likely due to the two-dimensional movement of the hyoid and the larynx during swallowing [[9]].

Head movements, either voluntary or involuntary, can severely influence the processing accuracy of dual-axis cervical accelerometry signals. For example, in previous contributions (e.g. [7]), the accuracy of automatic segmentation of such signals was severely diminished when participants performed so-called wet chin tuck maneuver, which involves tucking the chin towards the chest during each swallow. This postural technique is often used by speech-language pathologists to treat patients at risk of aspiration (entry of ingested food or liquids into the larynx below the level of the true vocal folds). Hence, for the further development of medical devices based on dual-axis cervical accelerometry signals, it is important to isolate the effects of head movements. In this paper, we study the effects of head motion, age and gender on the frequency content of dual-axis accelerometry signals, in the absence of swallowing activity.

## Methodology

### Data collection

Potential participants had to complete a short survey outlining their medical history. If an individual had any prior symptoms of swallowing difficulties, or had a history of stroke or other neurological conditions, head or neck cancer, neck or spinal injury or a tracheostomy, he/she was excluded from the study. In total, fifty consenting healthy adults (24 males) participated in this study ranging from 18 to 65 years of age (19 participants were 18-34 years old; 9 participants were 34-44 years old; 13 participants were 45-54 years old; and 9 participants were 55-65 years old). The research ethics board of Bloorview Kids Rehab (Toronto, Ontario, Canada) approved the study protocol.

**Figure 1:**
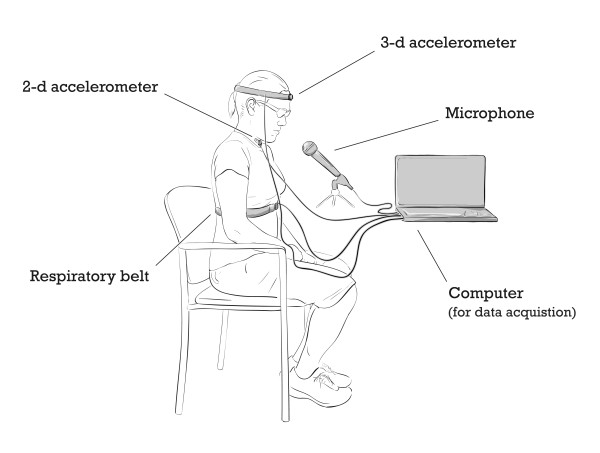
**Setup used in this experiment**. An experimental setup used in this study.

Upon the completion of the short survey, participants were seated comfortably in a chair for the rest of the experiment. To record cervical accelerometry signals (i.e., accelerometry signals without swallowing), a dual-axis accelerometer (ADXL322, Analog Devices) was placed on the neck of each participant anterior to the cricoid cartilage and secured with double-sided tape. The accelerometer has a measurement range of ± 5 *g*, a bandwidth of 2.5 kHz, a resonant frequency of 5.5 kHz, and sensitivity of 174 mV/*g*. A voltage supply (Model 1504, BK Precision) set at 5 V was used to power the acceleremoter. Furthermore, the two axes were positioned in the anterior-posterior (A-P) and superior-inferior (S-I) anatomical directions as shown in Figure 1. All participants were advised to refrain from swallowing during each task, but were permitted to swallow accumulated saliva between successive steps. No data were recorded during those swallows. Three additional sensors confirmed that the participants followed the data collection protocol properly. We collected signals from a triple-axis accelerometer (MMA7260Q, SparkFun Electronics) attached to a headband and centered on the participant's forehead to monitor head motions; a respiratory belt (1370G, Grass Technologies) secured around the participant's diaphragm to monitor breathing patterns; and a microphone placed 30 cm away from the participant's mouth to capture any vocalizations. The dual-axis accelerometer signals were filtered (passband 0.1-3000 Hz) and amplified in hardware (Grass P55 pre-amplifier). The subsequent signals were acquired at 10 kHz using a data acquisition card (NI USB-6210, National Instruments) and custom Labview software. The data were stored on a hard drive for subsequent analyses.

The data collection procedure included five primary tasks. Participants remained silent and were asked to:

1. tilt their head to the left side 10 times.

2. tilt their head to the right side 10 times.

3. tilt their head down 10 times.

4. tilt their head back 10 times.

5. rotate their head from right to left 5 times, and from left to right 5 times.

Participants performed each task only once, and the data collection did not generally last longer than 15 minutes per participant.

The participants also engaged in other tasks as part of the data collection protocol for a different study, hence, the additional sensors. For the current study, the additional sensors (head accelerometer, respiratory belt and microphone) simply served to confirm participant compliance with the experimental protocol. For example, head accelerometers were used to confirm that head motion was generated only when cued. Similarly, a microphone was used to ensure that participants did not vocalize during these tasks, while the respiratory belt was used to ensure that participants maintained normal breathing patterns during these tasks. Nevertheless, we did not engage in an extensive analysis of data collected using these sensors beyond a qualitative inspection to confirm participant compliance to the experimental protocol.

### Data analysis

The main goal of the data analysis was to understand the frequency content of dual-axis cervical accelerometry signals when head movements are present. Nonetheless, a few pre-processing steps were necessary to annul the effects of data acquisition equipment and to remove electrical noise. In particular, we first pre-processed the acquired signals with the inverse filters developed in [9] for the data acquisition instrumentation employed here. Secondly, the filtered signals were then denoised using the wavelet-based approach [10], which has previously proposed for cervical accelerometry signals [11]. Specifically, we implemented a 10-level discrete wavelet transform using the discrete Meyer wavelet with soft thresholding. To characterize the frequency content of these vibrations, three features commonly used in the analysis of audio and biomedical signals (e.g., [12,13]) were evaluated:

• peak frequency

(1) $$f_{p}=\;\underset{f\in \left[0,f_{\max}\right]}{\text{argmax}}|F_{x}(f){|}^{2}$$

• spectral centroid

(2) $$f_{c}=\frac{\int_{0}^{f_{\text{max}}}f|F_{x}(f){|}^{2}df}{\int_{0}^{f_{\text{max}}}|F_{x}(f){|}^{2}df}$$

• the bandwidth

(3) $$BW=\sqrt{\frac{\int_{0}^{f_{\text{max}}}{\left(f-f_{c}\right)}^{2}|F_{x}(f){|}^{2}df}{\int_{0}^{f_{\text{max}}}|F_{x}(f){|}^{2}df}\;}$$

where *F_x_*(*f*) represents the Fourier transform of the signal, and *f*_max _is the highest frequency available, i.e., half the sampling frequency.

Peak frequencies and spectral centroids are the most widely adopted indicators of spectral changes in signals [12], whereas the bandwidth denotes the power weighted average of the squared difference between the spectral components and the spectral centroid [13]. In other words, bandwidth represents spectral spread. For example, a signal consisting of a single sinusoid has a bandwidth of zero, while the bandwidth of white noise is in finite. In this paper, the concept of bandwidth also assists us in determining the presence of non-dominant signal components. These components may vary with anthropometric and demographic characteristics of the patient, and thus might be important the further development of a non-invasive swallowing aid.

To test for gender effects on the spectral parameters above non-parametric tests such as the Kruskal-Wallis (e.g., [14]) and the Mann-Whitney test (e.g., [15]) test were used, the latter with a Bonferroni adjusted significance level of *p *= 0.01.

## Results and Discussion

**Figure 2:**
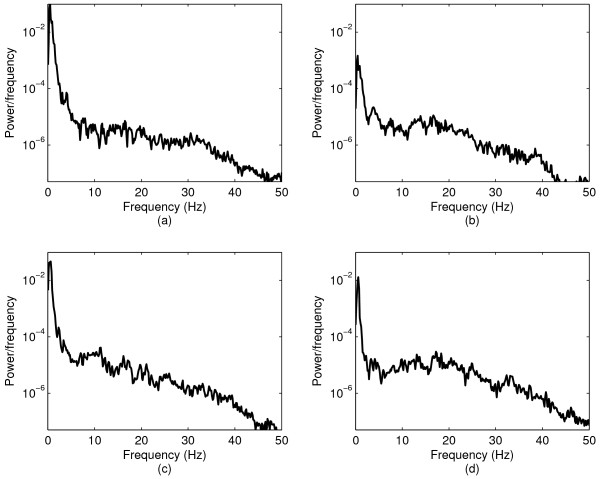
**Spectrums of sample signals during the third and fifth steps**. Spectrums of sample signals during the third and fifth steps: (a) and (b) depict spectrums of sample signals from the third step in the A-P and S-I directions, while (c) and (d) depict spectrums of sample signals from the fifth step in the A-P and S-I directions.

Tables 1, 2, 3 summarize the results of frequency analysis according to task, gender and age, respectively. Figure 2 depicts sample spectrums for illustrative purposes. Upon visual inspection, the head accelerometer data confirmed that participants only moved when cued. Likewise, the microphone and respiratory data verified, respectively, that participants neither vocalized nor breathed irregularly during the recording. Based on the presented results, several observations are in order. First, the observed peak frequency for each task can be attributed to large amplitude vibrations associated with head motions. Generally, greater variations in vibrations are observed in the S-I direction than in the A-P direction (e.g., [7,8]). Higher frequencies are observed for all 3 spectral features in the S-I direction (Mann-Whitney test, *p *< 0.01). However, when comparing these features on a task-by-task basis (e.g. comparing task 3 in the A-P direction with task 3 in the S-I direction), interesting results were obtained. A comparison of the peak frequencies for each task in the A-P and S-I direction revealed no statistical differences (Mann-Whitney test, *p *> 0.31). Similarly, the bandwidth for tasks 1, 2 and 5 exhibited no differences (Mann-Whitney test, *p *> 0.62). On the other hand, we obtained statistically different bandwidths for tasks 3 and 4 (*p *< 0.004) and the spectral centroids are statistically different for the A-P and S-I directions for each of the tasks (Mann-Whitney test, *p *< 0.01). Those latter differences were likely due to the differential effects of sagittal plane head motions on dual-axis accelerometry signals. Head motions in the sagittal place invariably involve greater motion in the anatomical S-I axis. Indeed, it has been previously noted that there is a larger signal variation in the S-I direction during the chin tuck maneuver, which is accompanied by significant excursion of the head downward towards the chest [7].

**Table 1:** Frequency analysis cross-tabulated by task and vibration axis.

	A-P direction			S-I direction		
	
Tasks	***f***_***p***_	***f***_***c***_	*BW*	***f***_***p***_	***f***_***c***_	*BW*
1. Tilt left	0.52 ± 0.19	6.62 ± 7.85	19.5 ± 4.33	0.59 ± 0.22	5.23 ± 3.74†	15.5 ± 5.06
2. Tilt right	0.53 ± 0.19	5.40 ± 5.99	17.9 ± 4.99	0.70 ± 0.37	6.58 ± 4.47†	20.1 ± 4.24
3. Tilt down	0.50 ± 0.11	2.12 ± 1.36	18.3 ± 6.73	0.61 ± 0.27	8.26 ± 5.06†	27.5 ± 7.86†
4. Tilt back	0.62 ± 0.25	1.69 ± 1.15	15.0 ± 3.16	0.64 ± 0.27	7.96 ± 6.96†	27.3 ± 4.22†
5. Rotate	0.57 ± 0.18	2.68 ± 1.85	29.2 ± 8.35	0.67 ± 0.22	6.88 ± 5.59†	31.4 ± 5.93
Overall	0.55 ± 0.18	3.70 ± 3.63	19.9 ± 18.5	0.64 ± 0.28	6.98 ± 5.15†	24.3 ± 21.1

Entries represent mean values plus/minus mean average deviations in Hz.

† Significantly different from the A-P direction

**Table 2:** Frequency analysis cross-tabulated by gender and vibration axis.

	A-P direction			S-I direction		
	
	***f***_***p***_	***f***_***c***_	*BW*	***f***_***p***_	***f***_***c***_	*BW*
Male	0.56 ± 0.14	3.73 ± 3.02	24.3 ± 14.4	0.85 ± 0.48	8.48 ± 5.15	32.5 ± 19.6†
Female	0.54 ± 0.13	3.68 ± 2.70	16.0 ± 8.60	0.60 ± 0.14	5.59 ± 2.11	16.8 ± 7.46

† Significantly different than female participants

**Table 3:** Frequency analysis cross-tabulated by age and vibration axis.

	A-P direction			S-I direction		
	
	***f***_***p***_	***f***_***c***_	*BW*	***f***_***p***_	***f***_***c***_	*BW*
*Age *< 35	0.54 ± 0.21	3.22 ± 1.89	10.4 ± 5.54	0.49 ± 0.12	6.47 ± 3.75	14.9 ± 6.90
35 ≤ *Age *< 45	0.47 ± 0.10	9.56 ± 14.3	32.6 ± 42.16	0.53 ± 0.12	4.06 ± 2.48	17.1 ± 17.1
45 ≤ *Age *< 55	0.49 ± 0.05	9.09 ± 12.5	26.2 ± 25.6	0.60 ± 0.17	10.6 ± 10.4	34.9 ± 35.1
*Age *≥ 55	0.68 ± 0.28	3.46 ± 2.13	35.9 ± 38.5	0.64 ± 0.16	10.5 ± 9.90	45.4 ± 58.2

Second, when all tasks are considered together, peak frequencies are statistically equivalent in both directions (Kruskal-Wallis test, *p *> 0.15). In other words, different head motions do not introduce different peak frequencies. However, the spectral centroids and bandwidths in both directions are statistically different (Kruskal-Wallis test, *p *< 0.01). This latter finding bears an important implication; while the vibrations associated with head motions are usually manifested through a dominant frequency in the spectrum, other non-dominant spectral components are also introduced. Third, statistically equivalent results were obtained for peak frequencies and spectral centroids in the A-P and S-I directions regardless of gender as shown in Table 2 (Mann-Whitney test, *p *> 0.31). Nevertheless, we found gender differences in bandwidths in the S-I direction (Mann-Whitney test, *p *< 0.01). In particular, male participants exhibited greater signal bandwidth, which can be attributed to their slightly higher (though not statistically different) peak frequencies and spectral centroids. Theoretically, there is little reason for the bandwidth of healthy participants to differ between genders (e.g., [16] and references within). Fourth, age did not influence any of the three considered spectral features as shown in Table 3 (linear regression analysis, *p *> 0.05).

### Implications

Methods relying on detrending non-stationary biomedical data (e.g., [17]) would be suitable in order to remove these low frequency components from dual-axis swallowing accelerometry signals.

The results bear important implications on the design of medical devices based on cervical accelerometry. In particular, gender and head motion need to be considered in algorithmic design. For example, an accelerometric device designed for dysphagia screening would likely require the specification of gender as an explicit input to its analytical algorithms. Furthermore, our findings suggest that the clinical protocol associated with a cervical accelerometry device should strictly limit head motions, since these motions greatly affect the processing accuracy of accelerometry signals (e.g. [7]). Conversely, accelerometric measurements made during postural maneuvers such as the chin-tuck, which entail head movements, ought to be interpreted with care.

## Conclusions

In this paper, we examined the spectral characteristics of cervical dual-axis accelerometry signals (i.e., dual-axis accelerometry signals without swallows) in the presence of head motions. In particular, we examined peak frequencies, spectral centroid and signal bandwidth while participants conducted five head motions. We found that the peak frequency is independent of sensor axis, age, gender and head motion task. However, spectral centroids were statistically different between the A-P and S-I direction for each task, and between tasks in a given direction. The bandwidth was also statistically different for tasks 3 and 4 between the A-P and the S-I directions. These differences showed that additional non-dominant frequency components were introduced to the accelerometry signals as a result of head motions. Also, the bandwidth was statistically different between male and female participants in the S-I direction, owing to the slightly higher peak frequencies and spectral centroids of male participants. Collectively, these findings suggest that the nature of head motion and patient gender should be considered in the analysis of dual-axis cervical accelerometry signals.

## Competing interests

The authors declare that they have no competing interests.

## Authors' contributions

ES conceived the study, analyzed the data, and drafted the manuscript. CMS conceived the study, and helped to interpret the results and draft the manuscript. TC conceived the study, and participated in the data analysis and manuscript writing. All authors read and approved the final manuscript.
